# Supplementary material for: Methodological steps used by authors of systematic reviews and meta-analyses of clinical trials: a cross-sectional study
Source: BMC Med Res Methodol. 2019 Jul 26;19:164. doi: 10.1186/s12874-019-0780-2 (PMC6659247; doi:10.1186/s12874-019-0780-2)
Supplement: Supplementary file 1 — How did authors extract and use data for systematic review and meta-analysis? (PDF 185 kb) [file 12874_2019_780_MOESM1_ESM.pdf]

BMC Medical Research Methodology

Methodological steps used by authors of systematic reviews and meta-analyses of clinical trials: A cross-sectional study

--Manuscript Draft--

**Manuscript Number:**	BMRM-D-17-00523R6
**Full Title:**	Methodological steps used by authors of systematic reviews and meta-analyses of clinical trials: A cross-sectional study
**Article Type:**	Research article
**Section/Category:**	Data analysis, statistics and modelling
**Funding Information:**	
**Abstract:**	Background<br>The quality of systematic reviews and meta-analyses (SR/MAs) depends on the extent of the methods used. We investigated the methodological steps used by authors of SR/MAs of clinical trials via an author survey.<br>Methods<br>We conducted an email-based cross-sectional study by contacting corresponding authors of SR/MAs that were published in 2015 and 2016 and retrieved through PubMed database. The 27-item questionnaire was developed to study the methodological steps used by authors when conducting a SR/MA and the demographic characteristics of the respondent. Besides the demographic characteristics, methodological questions regarding the source, extraction and synthesis of data were included.<br>Results<br>From 10,292 emails sent, 384 authors responded and were included in the final analysis. Manual searches were carried out by 69.2% of authors, while 87.3% do updated searches, 49.2% search grey literature, 74.9% use the Cochrane tool for risk of bias assessment, 69.8% assign more than two reviewers for data extraction, 20.5% use digital software to extract data from graphs, 57.9% use raw data in the meta-analysis, and 43.8% meta-analyze both adjusted and non-adjusted data. There was a positive correlation of years of experience in conduction of SR/MAs with both searching grey literature (P=0.0003) and use of adjusted and non-adjusted data (P=0.006).<br>Conclusions<br>Many authors still do not carry out many of the vital methodological steps to be taken when performing any SR/MA. The experience of the authors in SR/MAs is highly correlated with use of the recommended tips for SR/MA conduct. The optimal methodological approach for researchers conducting a SR/MA should be standardized.
**Corresponding Author:**	Nguyen Tien Huy, Ph.D.<br>Ton Duc Thang University<br>Ho Chi Minh, VIET NAM
**Corresponding Author E-Mail:**	nguyentienhuy@tdtu.edu.vn;tienhuy@nagasaki-u.ac.jp
**Corresponding Author Secondary Information:**	
**Corresponding Author's Institution:**	Ton Duc Thang University
**Corresponding Author's Secondary Institution:**	
**First Author:**	Hoang Thi Nam Giang
**First Author Secondary Information:**	
**Order of Authors:**	Hoang Thi Nam Giang
	Ali Mahmoud Ahmed
	Reem Fala
	Mohamed Magdy Khattab

Powered by Editorial Manager® and ProduXion Manager® from Aries Systems Corporation

	Mona Hassan Ahmed Othman
	Sara Abdelrahman
	Le Phuong Thao
	Ahmed Gabl
	Samar Elrashedy
	Peter N. Lee
	Kenji Hirayama
	Hosni Salem
	Nguyen Tien Huy, Ph.D.
**Order of Authors Secondary Information:**	
**Response to Reviewers:**	Dear Dr. Puljak,<br><br>We wish to thank you for carefully going through our manuscript and the constructive comments. We have revised the manuscript according to your suggestions. At this stage, we uploaded our manuscript as a clean version.<br><br>The individual contributions of all authors to the manuscript should be specified in the Authors' Contributions section. If there are any duplicated initials, please differentiate them to make it clear that the initials refer to separate authors.<br>Reply: We have amended Author's Contribution section as suggested.<br><br>Please add a "Conclusions" section after the "Discussion" section. This should state clearly the main conclusions of the research article and give a clear explanation of their importance and relevance.<br>Reply: We have added Conclusion section.<br><br>On behalf of all authors<br>Nguyen Tien Huy

Manuscript

Click here to access/download;Manuscript;68.2_Revision_Clean_20190613.

Click here to view linked References

Methodological steps used by authors of systematic reviews and meta-analyses of clinical trials: A cross-sectional study

Short title: Methodological steps used by systematic review authors

Hoang Thi Nam Giang[1,2,⊕], Ali Mahmoud Ahmed[2,3,⊕], Reem Yousry Fala[2,4], Mohamed Magdy Khattab[2,3], Mona Hassan Ahmed Othman[2,5], Sara Attia Mahmoud Abdelrahman[2,6], Le Phuong Thao[2,7], Ahmed Elsaid Abd Elsamie Gabl[2,8], Samar Ahmed Elrashedy[2,9], Peter N Lee[10], Kenji Hirayama[11], Hosni Salem[12], and Nguyen Tien Huy[13,14*]

[1]Faculty of Medicine and Pharmacy, The University of Da Nang, Viet Nam

[2]Online Research Club (http://www.onlineresearchclub.org/)

[3]Faculty of Medicine, Al-Azhar University, Cairo, Egypt

[4]Faculty of Medicine, Al-Azhar University, Damitta, Egypt

[5]Al-Azhar University, Faculty of Medicine for Girls (AFMG), Cairo, Egypt

[6]Ministry of Health and People-sector of Regional Affair, Cairo, Egypt

[7]University of Medicine and Pharmacy, Ho Chi Minh City, Viet Nam

[8]Faculty of Veterinary Medicine, Kafr Elshiekh University, Kafr Elshiekh, Egypt

[9]Faculty of Medicine, Tanta University, Tanta, Egypt

[10]P.N.Lee Statistics and Computing Ltd, 17 Cedar Road, Sutton, Surrey, SM2 5DA, UK

[11]Department of Immunogenetics, Institute of Tropical Medicine (NEKKEN), Leading Graduate School Program, and Graduate School of Biomedical Sciences, Nagasaki University, Nagasaki 852-8523, Japan.

[12]Faculty of Medicine, Cairo University, Cairo, Egypt

[13]Evidence Based Medicine Research Group & Faculty of Applied Sciences, Ton Duc Thang University, Ho Chi Minh City, 70000, Viet Nam

[14]Department of Clinical Product Development, Institute of Tropical Medicine (NEKKEN), Leading Graduate School Program, and Graduate School of Biomedical Sciences, Nagasaki University, Nagasaki 852-8523, Japan.

[⊕]Authors equally contributed to the work.

*Correspondence to:

Nguyen Tien Huy, Evidence Based Medicine Research Group & Faculty of Applied Sciences, Ton Duc Thang University, Ho Chi Minh City, Viet Nam

(E-Mail: nguyentienhuy@tdt.edu.vn)

Emails

Hoang Thi Nam Giang: htngiang@ud.edu.vn

Ali Mahmoud Ahmed: ali.m.ahmed@azhar.edu.eg

Reem Yousry Fala: Reemfalla@gmail.com

Mohamed Magdy Khattab: mohamed.magdy@azhar.edu.eg

Mona Hassan Ahmed Othman: monahassan3333@azhar.edu.eg

Sara Attia Mahmoud Abdelrahman: sarah1atteya@yahoo.com

Le Phuong Thao: lephuongthao294@gmail.com

Ahmed Elsaid Abd Elsamie Gabl: ahmedgabl.2014@gmail.com

Samar Ahmed Elrashedy: smar.ahmed1956@yahoo.com

Peter N Lee: peterlee@pnlee.co.uk

Kenji Hirayama: hiraken@nagasaki-u.ac.jp

Hosni Salem: dr_hosni@kasralainy.edu.eg

Nguyen Tien Huy : nguyentienhuy@tdt.edu.vn

ABSTRACT

Background

The quality of systematic reviews and meta-analyses (SR/MAs) depends on the extent of the methods used. We investigated the methodological steps used by authors of SR/MAs of clinical trials via an author survey.

Methods

We conducted an email-based cross-sectional study by contacting corresponding authors of SR/MAs that were published in 2015 and 2016 and retrieved through PubMed database. The 27-item questionnaire was developed to study the methodological steps used by authors when conducting a SR/MA and the demographic characteristics of the respondent. Besides the demographic characteristics, methodological questions regarding the source, extraction and synthesis of data were included.

Results

From 10,292 emails sent, 384 authors responded and were included in the final analysis. Manual searches were carried out by 69.2% of authors, while 87.3% do updated searches, 49.2% search grey literature, 74.9% use the Cochrane tool for risk of bias assessment, 69.8% assign more than two reviewers for data extraction, 20.5% use digital software to extract data from graphs, 57.9% use raw data in the meta-analysis, and 43.8% meta-analyze both adjusted and non-adjusted data. There was a positive correlation of years of experience in conduction of SR/MAs with both searching grey literature (P=0.0003) and use of adjusted and non-adjusted data (P=0.006).

Conclusions

Many authors still do not carry out many of the vital methodological steps to be taken when performing any SR/MA. The experience of the authors in SR/MAs is highly correlated with use of the recommended tips for SR/MA conduct. The optimal methodological approach for researchers conducting a SR/MA should be standardized.

Keywords: Systematic review, data extraction, meta-analysis, cross sectional study

Background

Systematic review (SR) with meta-analysis (MA) is a method for combining all available evidence fulfilling pre-determined criteria to answer a pre-defined question [1]. It is recognized as being a crucial component of the practice of evidence-based medicine in order to obtain the highest level of evidence to formulate recommendations for clinical practice [2].

Each systematic review with meta-analysis should be designed and planned carefully. It should involve a comprehensive method for extracting, combining and analyzing relevant data. A wrong conclusion can be drawn if the data were extracted and handled inappropriately or the MA is conducted using inappropriate methods [3].

SR/MA, as a growing field [4], may face methodological problems such as publication bias, which can affect the validity of a SR [3]. Moreover, when conducting a search, not all researchers use as many as possible of the relevant databases, and identify the related grey literature, i.e. reports not published in a journal or book [1]. Conference abstracts are recognized as being an important source of grey literature, and with the addition of other grey sources, account for approximately 10% of the literature included in SRs [1]. Depending on the research question, the importance of searching grey literature varies. Some SR/MAs rely more heavily on the inclusion of relevant grey literature than others.

The quality of a SR/MA depends not only on the number of databases searched, but also on the search strategy and search terms used. Also, SR/MA researchers may apply restrictions to the language and period of publication, which may lead to the loss of many potential studies, so affecting the final estimate [5]. Inappropriate data handling, including dealing with missing data, is still often detected, and can induce substantial bias [2]. The quality of a SR/MA depends on the methods used to minimize bias. There are more than 100 scales for assessing study quality or risk of bias, and authors should choose the appropriate metric carefully [6].

Using different quality scales can produce different results from the same study [7, 8]. Heterogeneity between the studies included in a MA, the so-called mixing of apples and oranges, represents another challenge that researchers face when pooling results from different studies [9]. Another major problem is that many researchers fail properly to interpret the results of their MAs [2].

While there may be no universal consensus adhered to by all authors of SRs, the methodology for conducting them is well developed and many guides for their conduct are available [1, 10, 11]. Notably, the Cochrane Handbook for Systematic Reviews of Interventions has defined procedures for addressing the methodological challenges in conducting SRs [1]. Apart from methodological guidelines, SR methodologists have developed and proposed reporting guidelines and quality assessment tools. A Measurement Tool to Assess Systematic Reviews (AMSTAR), AMSTAR-2, ROBIS, and Preferred Reporting Items for Systematic Reviews and Meta-Analyses (PRISMA) are the well-known tools to guide reporting [12-15].

Previous studies suggest that there may be considerable variability in the methodological processes authors choose to adopt while conducting a SR [16, 17]. In order to gain further insight, our cross-sectional survey aimed to investigate those methodological approaches used by authors when conducting SR/MAs of clinical trials.

Methods

Study design

We identified corresponding authors of SR/MAs that were published in 2015 and 2016 using the PubMed database. We retrieved 12,693 e-mail addresses using the search syntaxes ("systematic review" or "systematic literature review" or "meta-analysis" or (Cochrane Database Syst. Rev)) and (trial or trials or randomized or randomised) (See Appendix 1 for the search strategy). After removal of duplicate e-mails, starting in 26 September 2016, we sent out an e-mail asking respondents to complete a questionnaire. This was successfully

delivered to 10,292 authors, after excluding non-deliverable e-mails due to outdated e-mail addresses. A reminder was sent after one, three, and six weeks and the survey closed after 8 weeks (Figure 1) [18-20].

Questionnaire

The 27-item questionnaire was developed based on an analysis of the existing literature (Appendix 2). The questions concerning the demographic and professional characteristics of the authors included; age, gender, specialization, years of experience with SR/MA, number of published SR/MAs, highest impact factor of any published SR/MA, and experience in extracting data for a SR/MA. Further questions asked about the searching of databases, the risk of bias assessment, and data extraction for SR/MA, as well as about data synthesis and methods of MA. The questionnaire was designed as a Google form and a link was attached in the e-mail (https://goo.gl/4Dddpj). One response was set up for each participant. The validity of the questionnaire was tested by three waves of exploratory trials, in each of which the questionnaire was distributed to 30 colleagues who have experience with SR/MA.

Data analysis

Data were collected onto a Microsoft Excel spreadsheet and analyzed using the R Statistical Language (R Foundation for Statistical Computing, Vienna, Austria). Descriptive statistics, including frequencies and percentages in addition to means and standard deviations, were computed to describe respondents' characteristics and responses. Univariate logistic regression models were used to test associations with the professional background of the respondent (experience in conducting SR/MAs for more than five years, having more than fourteen published SR/MA papers, and having a SR/MA paper published in a journal with a journal impact factor (JIF) more than 10) as well as with practical attitudes to literature search, data extraction, and MA, with odds ratios (ORs) and 95% confidence interval (CIs) being presented. A two-tailed P value <0.05 was used to define a significant correlation.

Ethical considerations

The study strictly followed the Declaration of Helsinki [21]. We attached a cover letter to the email to inform participants about the survey objectives, that it would take approximately 10 minutes to complete the questionnaire, and that their participation in the study was voluntary. All participants were given information about the contact person and they had the opportunity to discuss issues related to the survey or the research project by giving a feedback. The invitation e-mail clearly stated that responses would remain anonymous and confidential. The first question of the survey represented an informed consent to join in the study. The questionnaire itself did not collect personal information. An ethical approval was not required for such an anonymous web-based survey, because Ton Duc Thang University, Ho Chi Minh city, Vietnam waives the need for ethical approval requests for such studies.

RESULTS

Demographic and professional characteristics

From the 10,292 e-mails sent, 385 responses were received. One response was omitted as most of the questions were incompletely answered, leaving 384 in the analysis shown in Table 1. The mean age of the participants was 44.0 ±11.6 and 258 (67.2%) were men. About half of the authors, 192 (50.7%), came from Europe, with internal medicine as the major specialization (49.0%). Among our respondents, there were 51 (13.5%) epidemiologists with some specialization in SRs. Over half the respondents (56.3%) had more than five years' experience in conducting a SR/MA. The mean number of published SR/MAs by our respondents was 13.6 ± 32.9. There were 56.5% of participants who had published SR/MAs in journals with an impact factor higher than 5. Most of the corresponding authors, 368 (95.8%), had experience in extracting data for MA.

Table 1 here

Searching information sources

As noted in Table 2, the majority, 241 (63.1%), of respondents searched three to five information sources, and nearly 45 information sources were mentioned as having been searched by at least one respondent. These included ProQuest dissertations and abstracts, conference proceedings, grey literature databases and thesis sources. The proportions of respondents searching each information source are presented in Figure 2. The information source most often searched was PubMed or MEDLINE (99.7%) followed by EMBASE (76.6%), Cochrane library (76.3%), the Web of Science (WoS) (44.3%), CINAHL (36.5%), SCOPUS (30.7%), Google Scholar (29.4%), and PsycINFO (27.3%).

Less than half of the respondents (n=171, 45%) always conducted a manual search, involving looking at references in relevant papers retrieved, in journal issues or in conference proceedings. As regards data extraction, 94.6% of respondents included all papers that appeared to be relevant. Also, 87.3% performed an updated search to include the most recent papers (Table 2). Significant positive associations were found between searching grey literature databases and whether researchers had experience in conducting SR/MAs for more than five years (OR=2.1, 95%CI [1.4 to 3.2], P<0.001) or had published more than 14 SR/MA publications (OR=2.6, 95%CI [1.5 to 4.2], P<0.001). There was no association between experience in conducting SR/MAs and either conducting a manual search or the number of information sources used in the search (Table 3).

Table 2 here

Table 3 here

Risk of bias assessment

There were 74.9% of the respondents who used the Cochrane tool to evaluate risk of bias in clinical trials. The remaining 25.1% reported using “other tools” and when asked to name those tools, the most frequent responses were: Downs and Black checklist, CONSORT, modified Jadad scale, and CAMARADES tool.

Data extraction

As regards data extraction, using two reviewers was the most common approach used (by 58.1%), then one reviewer extracting and another or more subsequently checking (30.2%), then using more reviewers (11.7%). Of the 376 respondents, 211 (56.1%) contacted the authors of the papers to obtain the original data if the data were represented only in figures and graphs. Only 77 researchers (20.5%) used software to extract data from figures and graphs. Other 13 researchers (3.5%) indicated that extraction data from figures is unreliable. There were 75 researchers (19.9%) who did not know of or use such digital software. The regression analysis (Table 3) indicated that authors with ≥ 14 publications in SR/MA were more likely to extract the data using two or more independent reviewers (OR=1.9, 95%CI [1.05 to 3.3], P=0.03). In addition, those authors and authors with SR/MA papers published in a journal with a JIF >10 more frequently used digital software to extract data from figures (OR=2.4, 95%CI [1.4 to 4.1], P=0.002) and (OR=2.3, 95%CI [1.3 to 4.0], P=0.003) respectively (Table 3).

Data synthesis

Occasionally, authors did not report the difference between pre- and post-intervention in both intervention and control groups in clinical trials. Instead, they separately reported the pre- and post-intervention data (pre/post). Of those 364 authors responding to that question, 161 (44.2%) reported previous knowledge about this and accounted for it in data extraction. Of these 161 researchers, 156 shared the way that they dealt with this data in the MA. Among these 156 authors, 25.6% used only post-difference values for each group, with 25% using pre- and post-difference values for each group with the correlation value. In addition, among the same 161 authors who have previous knowledge on pre/post data, 157 shared their practice when the only data available were the pre- and post-intervention values for each group without the correlation. Among these 157 authors, 53 (33.8%) chose to request the data from the

authors of the original articles. If there was no response from the original authors, 28 (17.8%) used a default correlation value of 0.5 and 32 (20.4%) conducted multiple analyses with correlation values ranging from 0.1-0.9. There were 44 authors (28%) who did not know what was meant by the correlation value.

Of the total of 384 authors, 382 shared their experience in using raw data in MA. Among the 382, 221 (57.9%) preferred pooling the raw data, while 26 (6.8%) preferred pooling the analyzed results. A total of 372 authors shared their experience in dealing with adjusted and unadjusted data. Among these, 58 (15.6%) meta-analyzed the unadjusted data alone, 118 (31.7%) meta-analyzed the adjusted data alone, and 163 (43.8%) meta-analyzed both.

One of the most controversial points when conducting a MA concerns repeating the control group data in subgroup MA, for example when the intervention group includes different doses, each with the same placebo control. Of the 384 responders, 350 answered the question on this. Of these, 275 (78.6%) do not do this because the same population cannot be pooled twice in one MA, and only the subtotal results should be meta-analyzed. In contrast, 75 (21.4%) wrongly pooled the same value twice in the same MA.

Regarding use of correlations in MA, 370 authors shared their experience in Pearson correlations, of whom 96 (25.9%) reported using it, while 368 authors shared their experience in Spearman correlations, of whom 71 authors (19.3%) reported using it. Of the total of 384 researchers, 190 answered the question regarding the combination of these two methods. Among these 190, 127 (66.8%) preferred using each method in two separate MAs, 23 (12.1%) preferred the combination of both methods, 34 (17.9%) use only Pearson's method, and 6 (3.2%) used only Spearman's method (Table 4).

There was a significant negative association between more than five-year experience in conducting SR/MA of the authors and pooling the same data twice in one MA (OR=0.3, 95%CI [0.2 to 0.7], P=0.004). compared with authors with lower experience, authors with

more than 14 published SR/MA papers significantly preferred to use the raw data in the MA rather than the analyzed data (OR=1.8, 95%CI [1.1 to 3.0], P=0.03). Conducting other MA procedures was not associated with experience in conducting SR/MA (Table 3).

Table 4 here

Discussion

Despite the presence of many guidelines and checklists concerning the methodology of SRs/MAs [12, 13, 22], the quality of published SR/MAs is variable and lacks consistency. [23-26]. Our study showed considerable variability in the methods that authors choose to adopt when conducting and reporting SR/MAs. Many authors still do not apply recommended methods. Thus, for example, authors fail to conduct manual searches for new studies, fail to update searches, carry out data extraction by only one reviewer, and report the same data twice in subgroup MA.

In our survey, the five most common information sources searched were PubMed, EMBASE, Cochrane library, WoS and CINAHL. Three of these five information sources (EMBASE, Cochrane library, CINAHL) corresponded to the most frequently searched databases in reviews of physiotherapy [27]. However, authors in our survey use databases that are freely available (PubMed) rather than those requiring a paid subscription (EMBASE). Besides its free availability, PubMed has updated several features to facilitate more comprehensive searching [27]. Conducting a comprehensive literature search is central to reducing selection and publication bias in SRs [28].

The number of databases and the quality of the search strategy are crucial for an effective literature search. In recent years, there is an increasing reliance on a range of databases or on the combination of different database including Medline and EMBASE, allied health databases (e.g., CINAHL and PsycINFO), and web-based searching to locate grey literature [29]. Some of the "databases" mentioned by the respondents are not exactly databases. This

indicates that not all SR authors have sufficient knowledge regarding search engines and information sources. Less than 50% of authors use manual searches, or “hand-searches”, based on reference lists of published papers and also possibly study relevant conference proceedings or specific journal issues [1, 30]. This is unsatisfactory as such additional searches are very important to retrieve reports missed from the electronic databases search and to overcome the problem of inadequate search strategies. Methods of manual searching can vary. Authors are not routinely expected to undertake manual searches of journal contents; however scrutinizing reference lists is recommended. One may expect to find significant correlations between searching grey literature or manual search and respondent characteristics indicating experience, such as having conducting SR/MA for more than five years, or having more than 14 SR/MA publications. A previous study concluded that grey literature searching, adjusting terms and author-reported searching in SRs were sub-optimal and need to be improved [31]. Also, this study suggested that librarian involvement contributes to a comprehensive and reproducible search strategy to study identification and helps to produce high quality SR/MAs [31]. Another study stated that searching for grey literature with the help of a librarian would be easier [32]. The involvement of other experts, including statisticians can also affect quality.

The tool used for quality assessment should cover all methodological criteria relevant to the validity and interpretation of the included papers, taking into consideration the design of the studies considered [33]. Several domains for detecting and controlling the risk and source of bias should be evaluated. In many SR/MAs of clinical trials, absence of allocation concealment and inadequate randomization and blinding were associated with overestimation of the effect. Pildal *et al*, who replicated a MA of 70 studies, found that more than two-thirds of papers, with an overall effect estimate favouring certain interventions, showed no significant effect estimate after excluding papers with inappropriate allocation concealment [34]. Among the different metrics, Cochrane proposed a robust tool for assessing risk of bias

of the included clinical trials [1, 35]. Although about 75.0% of the respondents used the Cochrane tool, we did not properly assess the usage of other metrics [25, 36, 37]. Most of the other tools mentioned by the authors are not risk of bias assessment tools, but tools for assessing the quality of reporting. Therefore, their usage for risk of bias assessment is inappropriate.

Data extraction and handling is a fundamental step, and one of those that most determines the reliability of a SR/MA. Although a third (30.2%) of our respondents considered that only one reviewer was needed to extract the data of interest, they used other reviewers to check the extracted data to avoid potential bias, a procedure which is considered acceptable by AMSTAR [38]. Jones *et al* analyzed the data extraction methods in 34 Cochrane reviews and reported that it was carried out by two extractors independently in 30, by only one extractor in two, with two not stating the number of extractors [39]. Recently, some software packages, such as Plot Digitizer and Getdata Graph Digitizer [40], have become available to extract data represented only in graphs. Using such software for extracting data from figures was faster and provided higher interrater reliability [41]. However, those software solutions have not yet been incorporated into methodological guidelines, so it was unsurprising to find that only 20.5% of authors used them.

Turning now to methods of conducting MA, a widespread barrier for computing and calculating effect sizes (when extracting data from studies) is when crucial data, such as variances, standard deviations and standard errors, are not available from the study [42]. To try to cope with this, a large diversity of conversions and alternative formulations of effect sizes are available, many offered as computer packages [43]. Lajeunesse *et al* outlined a few simple imputation approaches that can be used to fill gaps in missing SDs when conversions are not possible [44]. These approaches include relying on resampling approaches to fill gaps, and estimating the coefficient of variation from the (complete) observed data. These

approaches should only be applied when data extraction from all the studies has been completed. These SD imputation tools include metagear, which provides two variations on Rubin & Schenker's (1991) 'hot deck' imputation approach and imputes only SDs that are nearest neighbours relative to their means (i.e. it imputes SDs from data with means of a similar scale) [45]. Another SD imputation tool is Bracken's (1992) method for filling missing information using the coefficient of variation from all studies with complete information, which is a strictly random hot deck imputation [46].

Handling and analyzing pre/post continuous data remains a point for debate as the data in each study that should be pooled is the effect estimate, not the post data, and usually the correlation is not present. Regarding this point, the responses of the authors were similar for the proposed solutions. Not many respondents chose to contact the authors of the original reports to get the data. Contacting the authors does not occur frequently in reviews due to the low and delayed response rates [47]. However, 28% of the surveyed authors did not know what the correlation means, which may reflect their not having faced this issue before.

In MA, pooling the analyzed or estimated data is not recommended and may be misleading. In our case, only 6.8% used analyzed results. How to deal with adjusted or unadjusted data in MA is an issue that needs to be highlighted and further investigated. The percentage of authors who analyzed only adjusted data, only non-adjusted data, or both was 91.1%. Like meta-regression, subgroup MA is a method for testing the effect of covariates on the overall effect estimate. However, a common mistake is repeating the control group data in subgroup MA when the cause of subgrouping relates to the nature of the intervention group (such as different doses), which leads to hyperinflation of the control group in the overall effect size. Among our respondents, the percentage of authors who indicated they do that is 21.4%. When the primary studies reported a correlation, the pooled effect size is the correlation coefficient.

Therefore, it is satisfactory to find that two thirds of our respondents chose to use both Pearson and Spearman correlations in separate MAs.

A limitation of our study is the low response rate. However, since we contacted many potential participants, we still managed to get response from more than three hundred SR/MA authors. Many online surveys traditionally experience low response rates, which may result in selection bias and lower generalizability of results. Further, while SR/MA methodology has advanced in the years since Cochrane reviews first began to be published, our study did not choose to limit the search to reviews published since then.

Although we conducted piloting of the questionnaire, some of the questions may have still remained unclear to respondents. Furthermore, our questions did not cover some aspects, such as changing inclusion and exclusion criteria, or even changing the main study question after the search revealed an inadequate number of studies (sample size). Some Cochrane SRs have been published with zero studies included. Moreover, we did not ask any question about the composition of the SR/MA team, for example whether or not it included specialists like librarians and statisticians. We focused more on how each step was done. Similarly, we did not include a question about participation in Cochrane reviews to compare Cochrane authors with non-Cochrane authors. Furthermore, the absence of content analysis of published SRs is a limitation as it may provide more information compared to our survey. In the search strategy to identify SR/MAs in PubMed we did not choose to limit our search on publication date. This is one limitation of our study, as the methodology of SRs keeps evolving.

Conclusion

Many surveyed SR/MA authors indicated that they did not utilize many of the crucial methodological steps which should be considered in the conduct of an SR/MA. These insufficiently used methodological steps were: the manual and the updated search; data extraction by more than one reviewer independently; pooling the difference between both pre-

and post-treatment values in the MA; and avoiding including the same data twice in subgroup MA. The experience of the authors in doing SR/MAs is positively correlated with using the recommended procedures for conducting a SR/MA. Guidelines for optimal methodology for conducting SR/MAs remain to be defined and authors of such studies should be required to follow them. Journals should specify in their instructions for authors which methodological steps they expect to be reported in submitted SR/MAs

List of abbreviations

CI: confidence interval

JIF: journal impact factor

MA: meta-analysis

OR: odds ratio

SD: standard deviation

SR: systematic review

SR/MA: systematic review and meta-analysis

Declarations

Ethics approval and consent to participate

Participation by individuals capable of informed consent as subjects in this survey was voluntary and the informed consent was embedded in the cover letter of the email, an ethics approval from Nagasaki University not being required for an anonymous web-based survey. Written informed consent was obtained from participants by answering the first question of the survey (Q0. Have you read all the information about the study given in the cover letter and agreed to participate in this survey? - Yes/No).

Consent to publish

Not applicable

Availability of data and materials

The data will be made available on request

Competing interests

The authors declare that they have no competing interests.

Funding

No funding was received

Authors' Contributions

HTNG, AMA, KH, NTH, PNL collaboratively designed the study. All authors developed the conceptual framework for the manuscript. HTNG and AMA drafted the manuscript, which was then revised by NTH, KH, HS, RYF, MMK, MHAO, SAMA, LPT, AEAEG, SAE and PNL. All authors read and approved the final manuscript. All authors have agreed on personally accountable for the author's own contributions and to ensure that questions related to the accuracy or integrity of any part of the work were appropriately investigated.

Acknowledgements

We would like to thank all those who spent time to answer the questionnaire. Thanks to Online Research Club members (http://www.onlineresearchclub.org) for giving comment to improve the questionnaire. Thanks to Dr. Nguyen Lam Vuong (University of Medicine and Pharmacy, Ho Chi Minh City, Viet Nam) for giving valuable feedback on questionnaire.

REFERENCES

1. **Higgins JPT, Green S (editors). Cochrane Handbook for Systematic Reviews of Interventions Version 5.1.0 [updated March 2011]. The Cochrane Collaboration, 2011. Available from http://handbook.cochrane.org.**

2. Yuan Y, Hunt RH: **Systematic reviews: the good, the bad, and the ugly**. *The American journal of gastroenterology* 2009, **104**(5):1086-1092.
3. Esterhuizen TM, Thabane L: **Con: Meta-analysis: some key limitations and potential solutions**. *Nephrology Dialysis Transplantation* 2016, **31**(6):882-885.
4. Glass GV: **Primary, Secondary, and Meta-Analysis of Research**. *Educational Researcher* 1976, **5**(10):3-8.
5. Zoccali C: **Moderator's view: Meta-analysis: the best knowledge but not always shining gold**. *Nephrology Dialysis Transplantation* 2016, **31**(6):886-889.
6. Hung BT, Long NP, Hung le P, Luan NT, Anh NH, Nghi TD, Hieu MV, Trang NT, Rafidinarivo HF, Anh NK *et al*: **Research trends in evidence-based medicine: a joinpoint regression analysis of more than 50 years of publication data**. *PloS one* 2015, **10**(4):e0121054.
7. Sackett DL, Rosenberg WMC, Gray JAM, Haynes RB, Richardson WS: **Evidence based medicine: what it is and what it isn't**. *BMJ* 1996, **312**(7023):71.
8. Juni P, Witschi A, Bloch R, Egger M: **The hazards of scoring the quality of clinical trials for meta-analysis**. *Jama* 1999, **282**(11):1054-1060.
9. Balk EM, Bonis PA, Moskowitz H, Schmid CH, Ioannidis JP, Wang C, Lau J: **Correlation of quality measures with estimates of treatment effect in meta-analyses of randomized controlled trials**. *Jama* 2002, **287**(22):2973-2982.
10. Moyer A, Finney JW: **Rating methodological quality: toward improved assessment and investigation**. *Accountability in research* 2005, **12**(4):299-313.
11. Moayyedi P: **Meta-analysis: Can We Mix Apples and Oranges?** *The American journal of gastroenterology* 2004, **99**:2297.
12. Shea BJ, Grimshaw JM, Wells GA, Boers M, Andersson N, Hamel C, Porter AC, Tugwell P, Moher D, Bouter LM: **Development of AMSTAR: a measurement tool to assess the methodological quality of systematic reviews**. *BMC Medical Research Methodology* 2007, **7**(1):10.
13. Moher D, Liberati A, Tetzlaff J, Altman DG, Group P: **Preferred reporting items for systematic reviews and meta-analyses: the PRISMA Statement**. *Open medicine : a peer-reviewed, independent, open-access journal* 2009, **3**(3):e123-e130.
14. Shea BJ, Reeves BC, Wells G, Thuku M, Hamel C, Moran J, Moher D, Tugwell P, Welch V, Kristjansson E: **AMSTAR 2: a critical appraisal tool for systematic reviews that include randomised or non-randomised studies of healthcare interventions, or both**. *Bmj* 2017, **358**:j4008.
15. Whiting P, Savović J, Higgins JP, Caldwell DM, Reeves BC, Shea B, Davies P, Kleijnen J, Churchill R: **ROBIS: a new tool to assess risk of bias in systematic reviews was developed**. *Journal of clinical epidemiology* 2016, **69**:225-234.

16. Popay J, Roberts H, Sowden A, Petticrew M, Arai L, Rodgers M, Britten N, Roen K, Duffy S: **Guidance on the conduct of narrative synthesis in systematic reviews**. *A product from the ESRC methods programme Version* 2006, **1**:b92.
17. Mokkink LB, Terwee CB, Stratford PW, Alonso J, Patrick DL, Riphagen I, Knol DL, Bouter LM, de Vet HC: **Evaluation of the methodological quality of systematic reviews of health status measurement instruments**. *Quality of Life Research* 2009, **18**(3):313-333.
18. Andrews D, Nonnecke B, Preece J: **Electronic Survey Methodology: A Case Study in Reaching Hard-to-Involve Internet Users**. *International Journal of Human–Computer Interaction* 2003, **16**(2):185-210.
19. Cook C, Heath F, Thompson RL: **A Meta-Analysis of Response Rates in Web- or Internet-Based Surveys**. *Educational and Psychological Measurement* 2000, **60**(6):821-836.
20. **Schonlau, Matthias, Ronald D. Fricker, and Marc N. Elliott, Conducting Research Surveys via E-mail and the Web. Santa Monica, CA: RAND Corporation, 2002. https://www.rand.org/pubs/monograph_reports/MR1480.html. Also available in print form.**
21. **World Medical Association Declaration of Helsinki: ethical principles for medical research involving human subjects**. *Jama* 2013, **310**(20):2191-2194.
22. Moher D, Cook DJ, Eastwood S, Olkin I, Rennie D, Stroup DF: **Improving the quality of reports of meta-analyses of randomised controlled trials: the QUOROM statement. Quality of Reporting of Meta-analyses**. *Lancet* 1999, **354**(9193):1896-1900.
23. Dosenovic S, Kadic AJ, Vucic K, Markovina N, Pieper D, Puljak L: **Comparison of methodological quality rating of systematic reviews on neuropathic pain using AMSTAR and R-AMSTAR**. *BMC medical research methodology* 2018, **18**(1):37.
24. Sharma S, Oremus M: **PRISMA and AMSTAR show systematic reviews on health literacy and cancer screening are of good quality**. *Journal of clinical epidemiology* 2018, **99**:123-131.
25. Wu X, Sun H, Zhou X, Wang J, Li J: **Quality assessment of systematic reviews on total hip or knee arthroplasty using mod-AMSTAR**. *BMC Medical Research Methodology* 2018, **18**(1):30.
26. Shea B, Bouter LM, Grimshaw JM, Francis D, Ortiz Z, Wells GA, Tugwell PS, Boers M: **Scope for improvement in the quality of reporting of systematic reviews. From the Cochrane Musculoskeletal Group**. *The Journal of rheumatology* 2006, **33**(1):9-15.
27. Moseley AM, Elkins MR, Herbert RD, Maher CG, Sherrington C: **Cochrane reviews used more rigorous methods than non-Cochrane reviews: survey of systematic reviews in physiotherapy**. *Journal of clinical epidemiology* 2009, **62**(10):1021-1030.

28. Egger M, Juni P, Bartlett C, Holenstein F, Sterne J: **How important are comprehensive literature searches and the assessment of trial quality in systematic reviews? Empirical study**. *Health technology assessment (Winchester, England)* 2003, **7**(1):76.
29. Royle P, Waugh N: **Literature searching for clinical and cost-effectiveness studies used in health technology assessment reports carried out for the National Institute for Clinical Excellence appraisal system**. *Health technology assessment (Winchester, England)* 2003, **7**(34):iii, ix-x, 1-51.
30. Chapman AL, Morgan LC, Gartlehner G: **Semi-automating the manual literature search for systematic reviews increases efficiency**. *Health information and libraries journal* 2010, **27**(1):22-27.
31. Koffel JB: **Use of Recommended Search Strategies in Systematic Reviews and the Impact of Librarian Involvement: A Cross-Sectional Survey of Recent Authors**. *PloS one* 2015, **10**(5):e0125931.
32. Charrois TL: **Systematic Reviews: What Do You Need to Know to Get Started?** *The Canadian Journal of Hospital Pharmacy* 2015, **68**(2):144-148.
33. **Haynes RB. Clinical epidemiology: how to do clinical practice research: Lippincott williams & wilkins; 2012**.
34. Pildal J, Hrobjartsson A, Jorgensen KJ, Hilden J, Altman DG, Gotzsche PC: **Impact of allocation concealment on conclusions drawn from meta-analyses of randomized trials**. *Int J Epidemiol* 2007, **36**(4):847-857.
35. **Lajeunesse MJ, Koricheva J, Gurevitch J, Mengersen K. Recovering missing or partial data from studies: a survey of conversions and imputations for meta-analysis. Handbook of Meta-analysis in Ecology and Evolution 2013: 195-206.**
36. Dosenovic S, Jelicic Kadic A, Vucic K, Markovina N, Pieper D, Puljak L: **Comparison of methodological quality rating of systematic reviews on neuropathic pain using AMSTAR and R-AMSTAR**. *BMC Medical Research Methodology* 2018, **18**:37.
37. Sharma S, Oremus M: **PRISMA and AMSTAR show systematic reviews on health literacy and cancer screening are of good quality**. *Journal of clinical epidemiology* 2018, **99**:123-131.
38. **Noyes J, Lewin S. Chapter 5: Extracting qualitative evidence. In: Noyes J, Booth A, Hannes K, et al., eds. Supplementary Guidance for Inclusion of Qualitative Research in Cochrane Systematic Reviews of Interventions. Version 1 ed; 2011**.
39. Jones AP, Remmington T, Williamson PR, Ashby D, Smyth RL: **High prevalence but low impact of data extraction and reporting errors were found in Cochrane systematic reviews**. *Journal of clinical epidemiology* 2005, **58**(7):741-742.
40. **Zein H, Tran VL-H, Azmy A, et al. How to Extract Data from Graphs using Plot Digitizer or Getdata Graph Digitizer. 2015**.

41. Jelicic Kadic A, Vucic K, Dosenovic S, Sapunar D, Puljak L: **Extracting data from figures with software was faster, with higher interrater reliability than manual extraction**. *Journal of clinical epidemiology* 2016, **74**:119-123.

42. J. LM, R. FM: **Variable reporting and quantitative reviews: a comparison of three meta-analytical techniques**. *Ecology Letters* 2003, **6**(5):448-454.

43. **Del Re AC. compute. es: Compute effect sizes. R package version 2013: 0.2-.**

44. **Lajeunesse MJ, Koricheva J, Gurevitch J, Mengersen K. Recovering missing or partial data from studies: a survey of conversions and imputations for meta-analysis. Handbook of Meta-analysis in Ecology and Evolution 2013: 195-206**.

45. Rubin DB, Schenker N: **Multiple imputation in health-care databases: an overview and some applications**. *Statistics in medicine* 1991, **10**(4):585-598.

46. **Bracken MB. Statistical methods for analysis of effects of treatment in overviews of randomized trials. Effective care of the newborn infant 1992: 13-20**.

47. Schroll JB, Bero L, Gøtzsche PC: **Searching for unpublished data for Cochrane reviews: cross sectional study**. *BMJ : British Medical Journal* 2013, **346**.

Table 1. Demographic and professional characteristics of responders

**Characteristics**	**Number (%)**
Age (mean ± SD), n=360	44.0 ± 11.6
Men, n=384	258 (67.2)
European, n=379	192 (50.7)
Specialty, n=379	
Internal medicine	186 (49.0)
Surgery	94 (24.8)
SRs/statistics/epidemiology	51 (13.5)
Others	48 (12.7)
Year of experience in SR/MA, mean (SD), n=378	8.6 (6.3)
>5 years' experience	213 (56.3)
Number of publications in SR/MA, (mean ± SD), n=375	13.6 ± 32.9
Highest impact factor of published SR/MA, n=373	
0 – 2	34 (9.1)
2 – 5	128 (34.3)
5 -10	124 (33.2)
10 -20	49 (13.1)
>20	38 (10.2)
Experience in data extraction for SR/MA of clinical trials, n=384	368 (95.8)
Directly extracted	339 (88.3)
Instruct students to extract	29 (7.6)

The data is represented by the number and percentage (%) or the mean ± standard deviation (SD). SR/MA: systematic review and meta-analysis.

Table 2. The results of search databases, risk of bias assessment, and data extraction for SR/MA.

**Variable**	**Number (%)**
Number of databases used in SR/MA, n=382	
1 – 2	44 (11.5)
3 – 5	241 (63.1)
6 – 10	75 (19.6)
>10	22 (5.8)
Searched Grey Literature Databases, n=378	186 (49.2)
Conduct a manual search, n=380	
Always	171 (45.0)
Often	92 (24.2)
Sometimes	64 (16.8)
Seldom	35 (9.2)
Never	18 (4.7)
While extracting the data, you accidentally found a new relevant paper. Did you include this paper via manual search or other sources? n=371	
Yes	351 (94.6)
No	20 (5.4)
Did you update the search to get more recent papers, n=370	
Yes	323 (87.3)
No	47 (12.7)
Tools used to evaluate the risk of bias of clinical trials, n=351	
Cochrane Collaboration's tool	263 (74.9)
Other (Downs & Black, CONSORT, MODIFIED JADAD, CAMARADES TOOL, Pedro, GRADE….)	88 (25.1)
Number of reviewers in a team to extract the data, n=384	
One reviewer extracts it, another or more reviewers check it	116 (30.2)
Two reviewers extract it	223 (58.1)
Three reviewers extract it	20 (5.2)
Four or more reviewers extract it	25 (6.5)
The original articles give the data in only figures or graphical representation, n=376	
Contact authors to get raw data	211 (56.1)
I did not know there is a digital software to extract it	75 (19.9)
Use a digital software to extract it	77 (20.5)

I did not extract it because I think the digital software is unreliable	13 (3.5)
The data used in extracting the survival percentage. n=356	
Raw data	118 (33.1)
Percentage estimated from Kaplan-Meier curve	50 (14.0)
I have never analyzed it	177 (49.7)
Other	11 (3.1)

The data is represented by the number and percentage (%). SR/MA: systematic review and meta-analysis.

Table 3. Association of professional characteristics with good attitude in data extraction and MA procedures.

	Years of experience (>5 years)			Number of publications (>14)			Highest impact factor journals of published papers (>10)		
**Items**	OR	95% CI	P value	OR	95% CI	P value	OR	95% CI	P value
Number of databases used (>6 databases)	1	0.6-1.6	0.9	1.5	0.9-2.5	0.2	0.8	0.5-1.4	0.5
Search Grey Literature Databases	2.1	1.4-3.2	<0.001	2.6	1.5-4.2	< 0.001	1.3	0.8-2.0	0.4
Performing manual search	0.8	0.5-1.2	0.3	0.8	0.5-1.3	0.3	0.8	0.5-1.3	0.3
Update the search to get more papers	0.8	0.4-1.5	0.4	0.6	0.3-1.3	0.2	1.1	0.5-2.4	0.7
Number of reviewers to extract the data (≥2)	1.3	0.8-2.0	0.2	1.9	1.1-3.3	0.03	1.6	0.9-2.8	0.09
Using digital software to extract data from figures	1.6	0.9-2.7	0.07	2.4	1.4-4.1	0.002	2.3	1.3-4.0	0.003
Pooled twice in one MA	0.3	0.2-0.7	0.004	0.7	0.4-1.2	0.2	0.7	0.4-1.3	0.2
Using raw data in MA	1.5	0.99-2.26	0.05	1.8	1.1-3.0	0.03	1.2	0.8-2.0	0.4
Meta-analyze both adjusted and unadjusted data	0.8	0.5-1.2	0.3	0.9	0.5-1.5	0.7	1.0	0.6-1.7	0.8
Combined Pearson and Spearman one meta-analysis	0.99	0.4-2.4	0.98	1.2	0.4-3.3	0.7	0.7	0.2-2.1	0.5

OR; Odds ratio. CI; confidence interval. MA; Meta-analysis

Table 4. Results of MA and data synthesis

Variable	Number (%)
Know pre/post-intervention data and post-intervention data (Yes), n=364	161 (44.2)
Investigating the efficacy of an intervention, and the pre- and post-intervention (pre/post) values for each group with the correlation value were not available, n=156	
Used pre/post-difference values for each group	77 (49.4)
Used post values for each group	40 (25.6)
Used (pre/post) values for each group and use correlation value	39 (25.0)
If the (pre/post) values for each group are the only data available but the correlation value is not available, what did you do? n=157	
Request authors of original articles	53 (33.8)
Use a default correlation value of 0.5 if no response from authors	28 (17.8)
Analyze several tests with correlation values ranging from 0.1-0.9 if nonresponse from authors	32 (20.4)
I do not know what is meant by the correlation value	44 (28)
Using raw data (such as mean, SD) or analyzed results (such as p-values) in the MA, n=382	
Use raw data	221 (57.9)
Use analyzed result	26 (6.8)
It is dependent on case by case	135 (35.3)
MA of adjusted or non-adjusted data, n=372	
Unadjusted data alone	118 (31.7)
Adjusted data alone	58 (15.6)
Meta-analyze both ways	163 (43.8)
Select data that have higher level of nominal significance	9 (2.4)
I don’t know adjusted and unadjusted data	24 (6.5)
Repeating the same data of placebo in many subgroups when analyzing subgroup based on the concentration of the drug used, n=350	
Yes	75 (21.4)
No, because the same population cannot be pooled twice in one MA. So the author should only perform subtotal results.	275 (78.6)
Ever extracted Pearson correlation for analysis, n=370	96 (25.9)
Ever extracted Spearman correlation for analysis, n=368	71 (19.3)

Dealing with Pearson and Spearman correlation in the MA (combining or separating), n=190	
Analyze each method separately in two MAs	127 (66.8)
Use only Pearson method	34 (17.9)
Combine both in one MA	23 (12.1)
Use only Spearman method	6 (3.2)

The data is represented by the number and percentage (%). MA; meta-analysis; standard deviation

Figure legends:

1) Figure 1. The flow chart of our study explaining the steps of data collection, handling, and reporting
2) Figure 2: The proportions of respondents searching each database

Figure 1

Click here to access/download;Figure;Figure 1_Final.tif

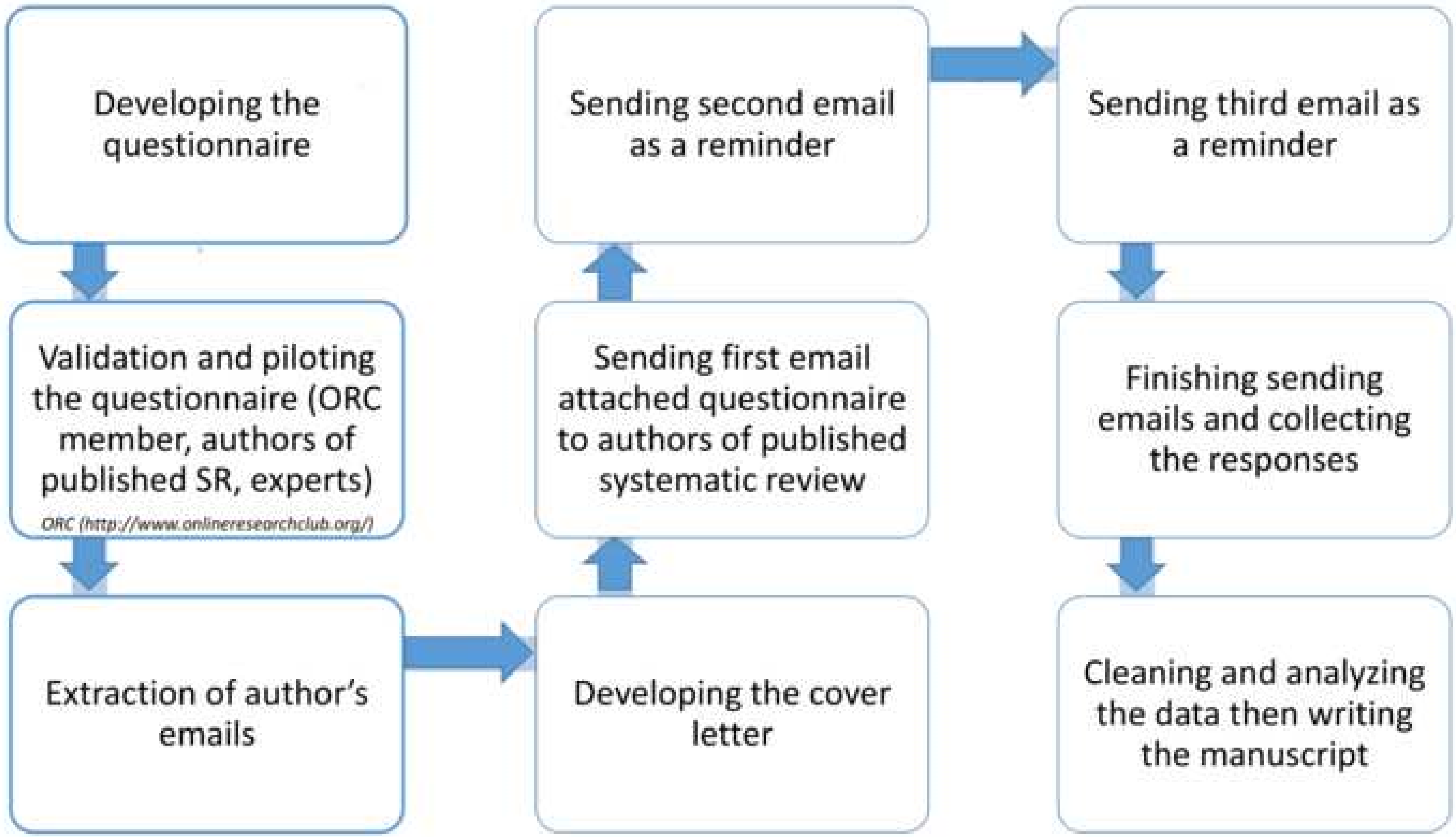

Figure 2

Click here to access/download;Figure;Fig-2.tif

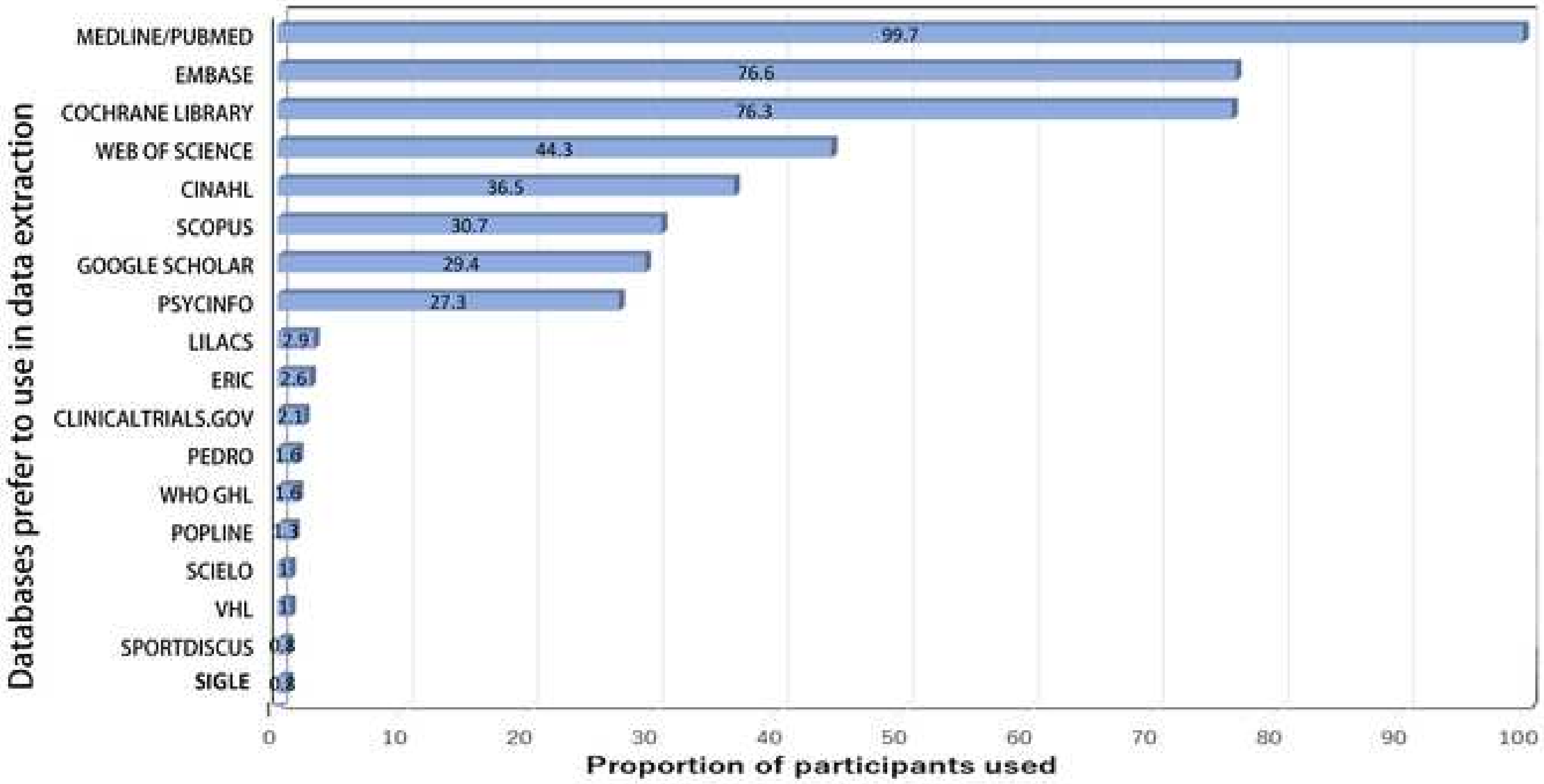

appendix 1

Click here to access/download
Supplementary Material
Appendix 1.docx

Appendix 2

Click here to access/download
Supplementary Material
Appendix 2.pdf
